# School-related mediators in social inequalities in smoking: a comparative cross-sectional study of 20,399 adolescents

**DOI:** 10.1186/1475-9276-8-17

**Published:** 2009-05-14

**Authors:** Christina W Schnohr, Svend Kreiner, Mette Rasmussen, Pernille Due, Finn Diderichsen

**Affiliations:** 1 Department of Social Medicine, Institute of Public Health, University of Copenhagen, Denmark; 2 Department of Biostatistics, Institute of Public Health, University of Copenhagen, Denmark; 3 National Institute of Public Health, University of Southern Denmark, Denmark

## Abstract

**Background:**

The aim of this study was to examine the associations between social inequalities and daily smoking among 13 and 15 year olds, and to determine the role of students' academic achievement and school satisfaction in these associations.

**Methods:**

HBSC is an international study including adolescents from 32 countries in Europe, Israel, and North America. The present study was based on information from 20,399 adolescents from Denmark, Sweden, Norway, Finland and the United Kingdom. Data were analysed by regression models.

**Results:**

The initial analyses showed significant inequality in daily smoking in all countries except for Sweden. When adjusted for the mediating role of academic achievement, estimates were attenuated, but remained significant in three countries.

**Conclusion:**

The study found social inequality in daily smoking in Denmark, Sweden, Norway, Finland and United Kingdom, as well as inequalities in students' academic achievement and school satisfaction. The analyses also showed that above average academic achievement was associated with lower OR of smoking. Teachers and politicians may find this information useful, and allocate resources to give higher priority to a supportive environment in schools especially for children and adolescents in lower social groups. Subsequently this prioritisation might contribute to reducing smoking in this group.

## Background

The growing recognition that social inequality in health and health behaviour is a global issue, and not only confined to affluent nations [1], has led to a demand for studies examining the mechanisms between socioeconomic status and health related outcomes. Disclosing the chain of associations from social position to an outcome related to health or health behaviour may contribute to a better understanding of these mechanisms and, in consequence, interventions may be designed to reduce health inequalities.

The literature on social inequality in health is voluminous, and one clear finding is that a major contributing cause of the inequality in mortality between social classes is smoking [2]. The literature on health inequalities among children and adolescents is more limited, even though the pattern is the same – lower socioeconomic position leads to poorer health and health behaviour regardless of the measure of socioeconomic position [3-8].

Multiple social factors have been shown to influence adolescent smoking [9]. In the period of transition between childhood and adulthood, adolescents are more susceptible to their surroundings and in particular the school, which constitutes a major social context in the lives of children and adolescents. Self reported academic achievement has been shown to play a role in the adoption of risk behaviors [10,11]. Other studies have reported that students' perceptions of school are related to socioeconomic position [5,12], academic achievement [13] and the link between educational achievement and adolescent substance use [14,15]. Furthermore, studies have shown that school influences may become more important over time, as influences in youth decrease [16].

When examining the literature on mechanisms generating social inequalities in adolescent risk behaviour, the evidence is relatively scarce. A study of Elgar and colleagues explored the contextual influences of income inequality on alcohol use [17], and another study by Kim and colleagues examined the impact of state, school and individual level factors on smoking behaviour, the latter study concluding that policies have the potential to influence smoking behaviour, but effect sizes were small [18]. In addition, a study by Piko and Fitzpatrick included smoking, drinking and marihuana use [6]; however, other than these few studies, research on associations in social inequality in adolescent smoking on an individual level may be considered uncharted territory. In order to be able to intervene against social inequalities in adolescent health behaviours such as smoking we need to identify modifiable factors that mediate the effect of socioeconomic position on health behaviour. This study deals with the question of whether or not academic achievement is such a mediator.

### Theoretical and structural framework

**Figure 1:**
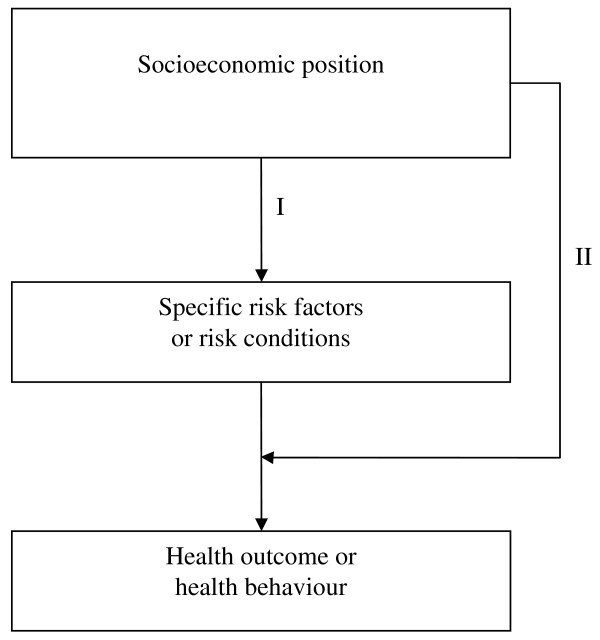
**A conceptual framework for studying health impact of socio economic position**. Adapted from Whitehead et al [20].

Diderichsen has proposed a framework [19] for the understanding of health inequalities. The framework delineates four main mechanisms: social stratification, differential exposure, differential susceptibility, and differential consequences. These mechanisms are used to explain how the social position and social context may play roles in generating the social patterning of disease and injury, and in the case of the present study, to examine the patterning of health risk behaviour measured by daily smoking. This framework has been used in comparative studies on mediating factors in health disadvantaged groups in Sweden and the United Kingdom [20]. The simplified version of the conceptual framework adapted from Whitehead (figure 1), shows these associations at an individual level. This line of enquiry considers how different social positions may carry different probabilities of being exposed to specific determinants or health hazards, the association or mechanism being denoted as differential exposure (figure 1, I). In addition, the health effect of a specific determinant may vary depending on the exposure to other interacting causes. This mechanism is denoted as differential susceptibility (figure 1, II). In line with this framework, the study analyses the mediating effect(s) of academic achievement in the social inequality of adolescent smoking.

West has emphasized the dynamics of health inequalities in adolescence denoted as equalisation [21,22]. The hypothesis on equalisation involves a change in social patterning from childhood characterized by inequality to youth characterized by relative equality. This could occur when effects associated with adolescence, the peer group and the youth culture cut across those of the family, home background and neighbourhood in such a way as to reduce class differences in health [21]. West points to effects associated with the secondary school [21], and in line with this framework, the present study analyses the potential effect modification of social position by academic achievement in relation to smoking, with the hypothesis that academic achievement buffers the effect of low social position on smoking.

## Methods

### The Health Behaviour in School-aged Children (HBSC) WHO Collaborative Study

HBSC is an international study of adolescents from countries in Europe, Israel, and North America, conducted in collaboration with the World Health Organization Regional Office for Europe. The study collects data on social and health indicators as well as health behaviors. The study provides comparable data on young people's health and lifestyle through the use of a common protocol.

The HBSC study consists of repeated cross-sectional cluster sampled surveys among 11-, 13- and 15-year-old school children in representative samples of approximately 1,500 students from each of the three age groups. The students fill in a standardized questionnaire during a school lesson after instruction from the teacher or researcher. HBSC has been collecting data on adolescents every fourth year since 1982. The 2001/02 HBSC study, on which the present paper is based, included 32 countries with a total of 162,323 students. Due to the relatively low proportion of daily smokers among 11-year olds, the analyses were conducted on 13- and 15-year olds only. Hence, the present study was based on 20,399 students from 5 countries in the HBSC survey (United Kingdom, Denmark, Sweden, Norway, and Finland), which were selected because of the relative comparability between school-systems and smoking patterns in these countries. Further methodological issues related to the HBSC as a cross-national survey are discussed in a recent paper by Roberts and colleagues [23].

### Determinant and outcome

It is a methodological challenge to define socio-economic status (SES) among children and adolescents, since indicators used for adults are inappropriate for use in research on these age groups [24]. HBSC has developed a summated scale, *Family Affluence Scale *(FAS), providing an indirect measurement of SES based on responses to four items: *Does your family own a car, van or truck? *(*1) No, 2) Yes, one, 3) Yes, more than one*), *Do you have your own bedroom for yourself? *(*1) No, 2) Yes*), *During the past 12 months, how many times did you travel away on holiday with your family? *(*1) Not at all, 2) Once, 3) Twice, 4) More than twice*), and *How many computers does your family own? *(*1) None, 2) One, 3) Two, 4) More than two*)

A previously published paper has described methodological challenges when comparing countries in the use of FAS [25]. In that paper, it was shown that responses to FAS items did not satisfy standard psychometric requirements of validity since items were locally dependent and function differentially. However, it was possible to fit a graphical log-linear Rasch model within which it was possible to equate FAS scores from different countries, different genders and different age groups to a common reference group. For details on these analyses we refer to Schnohr [25], and for the arguments supporting claims that FAS measurement is essentially valid and objective we refer to Kreiner and Christensen [26] and Kreiner [27].

The present study made use of the five-item FAS recommended by Schnohr [25], which includes the additional item *How well off do you think your family is?*(*1) Very well off, 2) Quite well off, 3) Average, 4) Not very well off, 5) Not at all well off*). The five items were weighed in a sum scale of 0 to 9, with the FAS means and standard deviations (SD) being shown in table 1. For further information on FAS, readers are recommended to read a recent paper published by Currie and colleagues [28].

**Table 1:** Data characteristics – included variables

	No. of students	% daily smokers	Mean FAS (SD)	Academic achievement (%)		Liking school (%)	
				
				Very good/good	Average/below average	Like a lot/like a bit	Don't like very much/don't like at all
13-year olds							

Denmark	1.519	2.8	7.29 (1.37)	64.9	35.1	76.1	23.9

Finland	1.641	6.5	5.87 (1.98)	56.8	43.2	58.8	41.2

Norway	1.699	3.6	7.14 (1.53)	63.5	36.5	89.9	10.1

Sweden	1.139	3.4	7.24 (1.45)	67.4	32.6	77.4	22.6

United Kingdom	4.651	6.7	6.56 (1.74)	67.0	33.0	70.6	29.4

15-year olds							

Denmark	1.344	14.4	7.42 (1.39)	55.4	44.6	72.7	27.3

Finland	1.699	22.8	5.84 (1.94)	49.6	50.4	50.2	49.8

Norway	1.604	17.6	7.18 (1.60)	58.5	41.5	83.0	17.0

Sweden	1.200	9.6	7.24 (1.41)	58.5	41.5	71.2	28.8

United Kingdom	3.903	16.5	6.59 (1.69)	63.9	36.1	61.9	38.1

Smoking was measured by the question *How often do you smoke tobacco at present?*(*1) Every day, 2) At least once a week, but not every day, 3) Less than once a week, 4) I do not smoke*). Daily smoking was used since the alternatives (less than weekly, have tried etc.) cover a broader range of use, and daily use is seen as a stable use, which would reduce misclassification as much as possible.

### School environment: Mediator and confounder

Two variables on relation to the school environment have been used in all the included HBSC countries. Academic achievement was included as a mediator and effect modifier in the association between FAS and daily smoking. Academic achievement was measured by *In your opinion, what does your class teacher(s) think about your school performance compared to your classmates?*(*1) Very good, 2) Good, 3) Average, 4) Below average*). This variable was dichotomized into adolescents experiencing themselves as *very good/good *in one category and *average/below average *in another category. School satisfaction was measured by *How do you feel about school at present? *(*1) I like it a lot, 2) I like it a bit, 3) I don't like it very much, 4) I don't like it at all*), which was included as a potential effect modifier and confounder as well as age and gender. The HBSC protocol provides more detailed information about the variables included in the study [29].

### Statistical analysis

**Figure 2:**
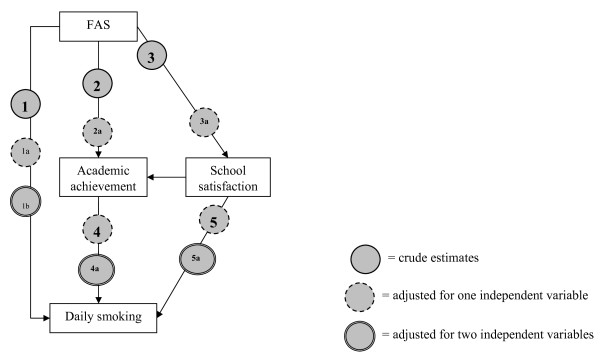
**Mechanistic associations in the study**.

The initial step of data analysis involved descriptive statistics on determinant, outcome, mediating and confounding variables. Binary logistic regression was then done on the association between FAS and daily smoking adjusted for age and gender. We examined possible interaction terms as a test for effect modification in a multiplicative model. Final analyses were conducted by use of multiple regression analyses of the associations illustrated in figure 2, all adjusted for age and gender.

*Differential susceptibility *and the buffering effect of school environment were tested by calculating interaction terms in the logistic regression analyses. Analyses were stratified by country.

## Results

The number of students included in the study ranged from a total of 2,339 in Sweden to 8,554 in the United Kingdom. Age and gender specifications are shown in table 1. The proportion of daily smokers varied depending on age. Among the youngest age group (13–14-year olds), the lowest overall prevalence was seen in Denmark, with a bit less than 3% daily smokers. The highest prevalence was found among girls in the United Kingdom (8.4%), and the highest overall prevalence was observed in Finland with 6.6% daily smokers among boys and 6.3% daily smokers among girls. Among the older students (15–16-year olds), Sweden had the lowest prevalence with 5.7% among boys and 13.5% among girls, and Finland had the highest prevalence with 22.1% among boys and 23.5% among girls (table 1).

Mean of the Family Affluence Scale (FAS) for the entire study group was 6.74 (SD = 1.74) indicating relatively high affluence in the selected countries. The lowest mean was observed in Finland and the highest in Denmark.

Self reported academic achievement was relatively constant across the five countries. Five per cent considered them *below average *and 30–40% thought they were *average *when compared to their classmates, and more than half (55–60%) thought they were *above average *(table 1).

The variable on school satisfaction had remarkable variation. In Norway, respondents were most satisfied, with an overall percentage of 87% with high school satisfaction *a bit *or *a lot*. In Finland, respondents were most dissatisfied, with 54% of the sample with high school satisfaction. The proportions of children with low school satisfaction varied from 15% to 45% across the five countries, and between 7% to 37% were happy with their school (table 1).

Interaction terms were tested for all covariates, but only the interaction between country and FAS in its effect on smoking was significant (p < 0.0001). Analysis is therefore stratified by country. This interaction is also illustrated by model 1, where estimates differ among countries (figure 1).

The mediation question was dealt with in a series of analyses on the models shown in table 2 and illustrated by figure 1. The crude association between FAS and daily smoking was clear, and OR varied between 0.27 (0.15–0.50) in Norway to 0.43 (0.16–1.16) in Sweden, indicating increased odds for adolescents in lower FAS to be daily smokers (figure 1).

**Table 2:** Logistic regression adjusted for age and gender (OR (95% CI))

Country	Model 1	Model 2	Model 2a	Model 3	Model 3a	Model 4
	FAS on smoking	FAS on academic achievement	FAS on academic achievement adjusted for school satisfaction	FAS on school satisfaction	FAS on school satisfaction adjusted for academic achievement	Academic achievement on smoking adjusted for FAS
Denmark	0.37 (0.16–0.85)	3.69 (2.25–3.06)	3.26 (1.96–5.43)	3.02 (1.77–5.16)	2.21 (1.27–3.84)	0.35 (0.26–0.46)

Sweden	0.43 (0.16–1.16)	3.43 (2.02–5.84)	2.61 (1.51–4.53)	5.50 (3.11–9.73)	4.37 (2.43–7.87)	0.43 (0.31–0.61)

Norway	0.27 (0.15–0.50)	3.55 (2.36–5.33)	2.93 (1.93–4.45)	5.59 (3.28–9.52)	4.12 (2.37–7.18)	0.30 (0.23–0.38)

Finland	0.35 (0.22–0.54)	3.76 (2.72–5.20)	3.40 (2.42–4.78)	2.12 (1.53–2.93)	1.56 (1.11–2.19)	0.21 (0.17–0.26)

United Kingdom	0.34 (0.24–0.48)	2.18 (1.73–2.76)	1.85 (1.45–2.37)	2.11 (1.67–2.68)	1.78 (1.39–2.27)	0.33 (0.29–0.38)

						

	Model 4a		Model 5	Model 5a	Model 1a	Model 1b
	Academic achievement on smoking adjusted for FAS and school satisfaction		School satisfaction on smoking adjusted for FAS	School satisfaction on smoking adjusted for FAS and academic achievement	FAS on smoking adjusted for academic achievement	FAS on smoking adjusted for acad.ach. and school satisfaction

Denmark	0.42 (0.31–0.58)		0.34 (0.26–0.46)	0.42 (0.31–0.56)	0.47 (0.20–1.10)	0.53 (0.22–1.26)

Sweden	0.52 (0.37–0.74)		0.33 (0.23–0.46)	0.39 (0.27–0.55)	0.54 (0.20–1.50)	0.78 (0.28–2.16)

Norway	0.31 (0.24–0.40)		0.53 (0.40–0.71)	0.69 (0.51–0.93)	0.37 (0.19–0.69)	0.41 (0.22–0.78)

Finland	0.24 (0.19–0.31)		0.37 (0.30–0.46)	0.51 (0.41–0.64)	0.55 (0.34–0.87)	0.59 (0.36–0.95)

United Kingdom	0.42 (0.36–0.49)		0.45 (0.30–0.40)	0.45 (0.39–0.52)	0.39 (0.27–0.56)	0.42 (0.29–0.60)

The association between FAS and academic achievement showed that children with low FAS more often considered themselves *average *or *below average *when compared to their classmates, with the lowest OR of 2.18 (1.73–2.76) found in the United Kingdom, and the other countries having estimates somewhat higher at around 3.5 (model 2). When including school satisfaction as a covariate in the association between FAS and academic achievement (model 2a) the OR was attenuated, but the confidence interval (CI) remained below 1 indicating a significant association. Model 3 demonstrates the crude association between FAS and school satisfaction, which showed a strong OR of around 5.5 in Sweden and Norway, and just a bit more than 2 in Finland and the United Kingdom (model 3). When adjusting for academic achievement in the association between FAS and school satisfaction, a similar attenuation was observed as between model 2 and 2a. A steep association remained between FAS and school satisfaction, in particular for Norway and Sweden, with an OR of 4.37 (2.43–7.87) for the latter (model 3a). Model 4 shows the association between academic achievement and daily smoking, with an inverse relation, this relation being strongest in Finland with an OR of 0.21 (0.17–0.26), and weakest – however still strong – in Sweden where the OR was 0.43 (0.31–0.61) (model 4). Including school satisfaction in this model did not alter the estimates noticeably (model 4a). As an intermediate analysis the association between school satisfaction and daily smoking was assessed with and without inclusion of academic achievement, and the estimates were similar to those of model 4 and 4a, and showed an inverse relation between school satisfaction and daily smoking, which was highest in Norway and lowest in Sweden.

Models 5 and 5a reveal an inverse relation between school satisfaction and daily smoking with and without adjustment for academic achievement. Model 1a tests the mediation hypothesis by adding academic achievement to model 1; OR values were closer to 1 indicating that some of the observed effect of FAS was indirectly mediated by academic achievement.

When comparing models 1 and 1a, a general increase in OR was observed, indicating that the effect of FAS on daily smoking may be partly explained by the included variables. The OR went from 0.37 to 0.47 for Denmark, from 0.43 to 0.54 for Sweden, from 0.27 to 0.37 for Norway, from 0.35 to 0.55 for Finland, and from 0.34 to 0.39 for the United Kingdom. When the mediator, academic achievement, was introduced into the model, the excess odds (OR-1) was reduced by 15% to 30%. The change in OR was particularly weak in the United Kingdom, which could be due to the weaker effect of FAS on academic achievement here.

The hypothesis that the school environment (measured as school satisfaction and academic achievement) modifies the effect of FAS [21] or that FAS modifies the effect of academic achievement [19] was tested as an interaction (departure from multiplicativity) in the model. There was neither interaction across the countries nor in the pooled analysis. Departure form additivity was not tested.

## Discussion

This study found inequalities in daily smoking among adolescents in Denmark, Sweden, Norway, Finland and the United Kingdom, with the association being partially mediated by academic achievement, since the OR was attenuated after adjusting for academic achievement. The reduction found was most pronounced in Finland, where the lowest school satisfaction was found. Students from Finland score highest in the PISA study [30]. This fact, combined with the finding that adolescents from Finland generally were the least satisfied with the school, can lead to the speculation that Finnish school systems pose high demands on students. The reduction was least pronounced in the United Kingdom, where there was also a generally low level of relative social inequality in academic achievement.

It is reasonable to handle academic achievement as a mediator since it can not be a confounder due to no possible influence on FAS. For school satisfaction the causal pattern is more complicated. School satisfaction seems certainly to be an effect of FAS and a determinant of smoking. But it might be both a determinant to and an effect of low academic achievement, and therefore a confounder of the effect of academic achievement on smoking.

Potential confounding of the effect of academic achievement on smoking other than by FAS and liking school, is also plausible, and might bias the estimated mediation fractions.

The insignificant association between FAS and smoking found in Denmark and Sweden may be due to the low prevalence of smokers, so that the conclusions drawn in this study were based on the tendencies from the OR, which was 0.53 (95% CI: 0.22–1.26 for Denmark and 0.78 (95% CI: 0.28–2.16) for Sweden (model 1b).

The lack of interactions gives no support for the hypothesis on *differential susceptibility *to the effect of school achievement (figure 1, II), nor for the hypothesis on *equalisation*-effect of school environment.

The mediating role of academic achievement emphasizes the role of teachers in supporting students from deprived families. If this is done with a focus on students from lower socio-economic positions, it might help reduce the social inequality in smoking prevalence.

There are a number of weaknesses in the present study. The cross-sectional nature of the survey does not make optimal room for empirical analyses of mechanistic associations, but only for testing if patterns of associations are consistent with hypotheses. Furthermore, there are possibilities of misclassification and common method bias, since data are based on self-reported questionnaires, and systematic differences in smoking or school satisfaction, for example, may be due to social position. A variable such as smoking tends to be underestimated, a precaution we have tried to comply with by including this variable in its most stable category, *daily smoking*. The present study has also not included a number of known determinants of adolescent smoking such as family smoking behaviors or peer smoking. These variables are not collected in the international HBSC survey, and a consequence might be that analyses are biased by some residual confounding.

The strength of the study is the dataset, which is nationally representative and comparable across countries. The data provide an opportunity to analyse differences in adolescents' health behaviours, and could potentially provide useful knowledge on the associations generating social inequalities in health among adolescents, an area that has not received the same attention as similar research on adults. Earlier longitudinal studies of Koivusilta and colleagues have found that health behaviours and educational tracks have a strengthening influence on each other in the developmental process [31], which may indicate that it is a difficult task to separate these variables and empirically test their independent associations.

The study found social inequalities in adolescent smoking behaviours across five European countries, and identified academic achievement as a mediator in the association. This finding supports the assumption that doing well in school may prevent smoking. The findings were consistent even though the group under study was adolescents, for whom several studies have shown that social inequality is less pronounced, when compared to children and adults [8,22,23]. Earlier studies have even shown mixed effects for risk behaviours and variations between genders [32]. Despite these particular challenges around adolescents, study of this age group will continue to be an area of public health interest, since the prevention of smoking among adolescents has great potential in a wider health-promoting context.

## Conclusion

The analyses showed social inequalities in daily smoking among adolescents, and also that academic achievement in school is an important mediator of the effect of family social position on smoking. Teachers and politicians may find this information useful, and allocate resources to giving a higher priority to a supportive environment in schools, especially for children and adolescents in lower social groups. Subsequently this resource allocation might contribute to improved public health and reduced health inequalities.

## Abbreviations

HBSC: Health Behavior in School-aged Children; WHO: World Health Organization; UK: United Kingdom; SES: Socio-economic status; FAS: Family Affluence Scale; OR: Odds Ratio; CI: Confidence interval; SD: Standard deviation.

## Authors' contributions

CS performed the statistical analyses, drafted and finalized the manuscript, SK assisted the statistical analyses, MR and PD contributed to the manuscript and with references, FD supervised analyses and commented the manuscript at several stages. All authors have read and approved the final manuscript.
